# A Field Test of the NORMAL Job Aid With Community Health Workers in Kenya to Address Contraceptive-Induced Menstrual Changes

**DOI:** 10.9745/GHSP-D-22-00364

**Published:** 2023-02-28

**Authors:** Holly M. Burke, Alfayo Wamburi, Kate H. Rademacher, Christina Misa Wong, Eunice Were, Emily Hoppes, Marsden Solomon

**Affiliations:** a FHI 360, Durham, NC, USA.; b FHI 360, Nairobi, Kenya.; c Consultant, Nairobi, Kenya.

## Abstract

**Introduction:**

Concerns about contraceptive-induced menstrual changes (CIMCs) contribute to nonuse and discontinuation of family planning (FP). Current counseling materials inadequately address these concerns. After obtaining initial feedback, we field-tested an adapted version of the NORMAL job aid that guides community health workers on how to counsel women about CIMCs. The field test aimed to help understand how the job aid was used, the challenges faced in using it, and recommendations to improve the job aid.

**Methods:**

Sixteen community health volunteers (CHVs) from 2 subcounties in Kenya were trained on the 2-page job aid and given copies of the job aid to use with clients. Six to 8 weeks after the training, we interviewed the CHVs about their experiences using the job aid. The interviews were audio-recorded, transcribed, and analyzed to identify qualitative themes.

**Results:**

All 16 CHVs reported using the job aid each time they counseled. All liked it and said they used it because it gave them new information and made counseling easier. All reported offering the job aid to most clients and that most clients accepted a copy. CHVs noted clients referred their friends and family to them after counseling using the job aid. CHVs said the job aid reduced clients’ and their male partners’ concerns about CIMCs and helped clients select or switch FP methods. Most CHVs did not have suggestions for improving the job aid.

**Conclusion:**

The job aid was highly acceptable to the CHVs who participated in this small assessment. According to the CHVs, it increased counseling effectiveness and may help increase uptake and continued use of FP methods directly through counseling or indirectly through diffusion in the community. Though further research is needed in other settings and to quantify its impact, we recommend this promising job aid be adapted for wider use.

## Abstract (precis)

A job aid for counseling clients on contraceptive-induced menstrual changes shows potential to improve counseling effectiveness, clients’ uptake, and continued use of hormonal contraceptive methods and the copper intrauterine device.

## Teaser

**Key Findings:**

- An adapted, 2-page job aid for counseling clients on contraceptive-induced menstrual changes was found to be highly acceptable among a small sample of community health volunteers in 2 subcounties in Kenya.
- According to the community health volunteers, the job aid increased the effectiveness of their counseling and supported diffusion of information in the community.

**Key Implication:**

- Improved counseling on contraceptive-induced menstrual changes may increase uptake and continued use of family planning methods. Policymakers and managers should consider introducing the NORMAL job aid after additional evaluation with the intended audiences.

## INTRODUCTION

Use of contraception—including hormonal methods and nonhormonal intrauterine devices (IUDs)—often results in contraceptive-induced menstrual changes (CIMCs). Common CIMCs include changes in bleeding duration, volume, frequency, and/or regularity, as well as changes in uterine cramping and pain. In addition, CIMCs often change over time with continued contraceptive method use; for example, a user might experience heavier bleeding when they begin using a method but then later become amenorrheic.^1^^,^^2^ Evidence indicates fears, misconceptions, or negative experiences related to CIMCs among users often contribute to discontinuation and nonuse of family planning (FP) methods. Common fears include that CIMCs can lead to negative health consequences, including infertility.^3^^,^^4^ At the same time, reduced bleeding or amenorrhea (paused bleeding) can have important noncontraceptive health and lifestyle advantages (e.g., ability to engage in work or education activities) for users,^5^^,^^6^^,^^7^ and a growing body of evidence suggests that these changes may be desirable for some.^8^^,^^9^^,^^10^

**FIGURE 1:**
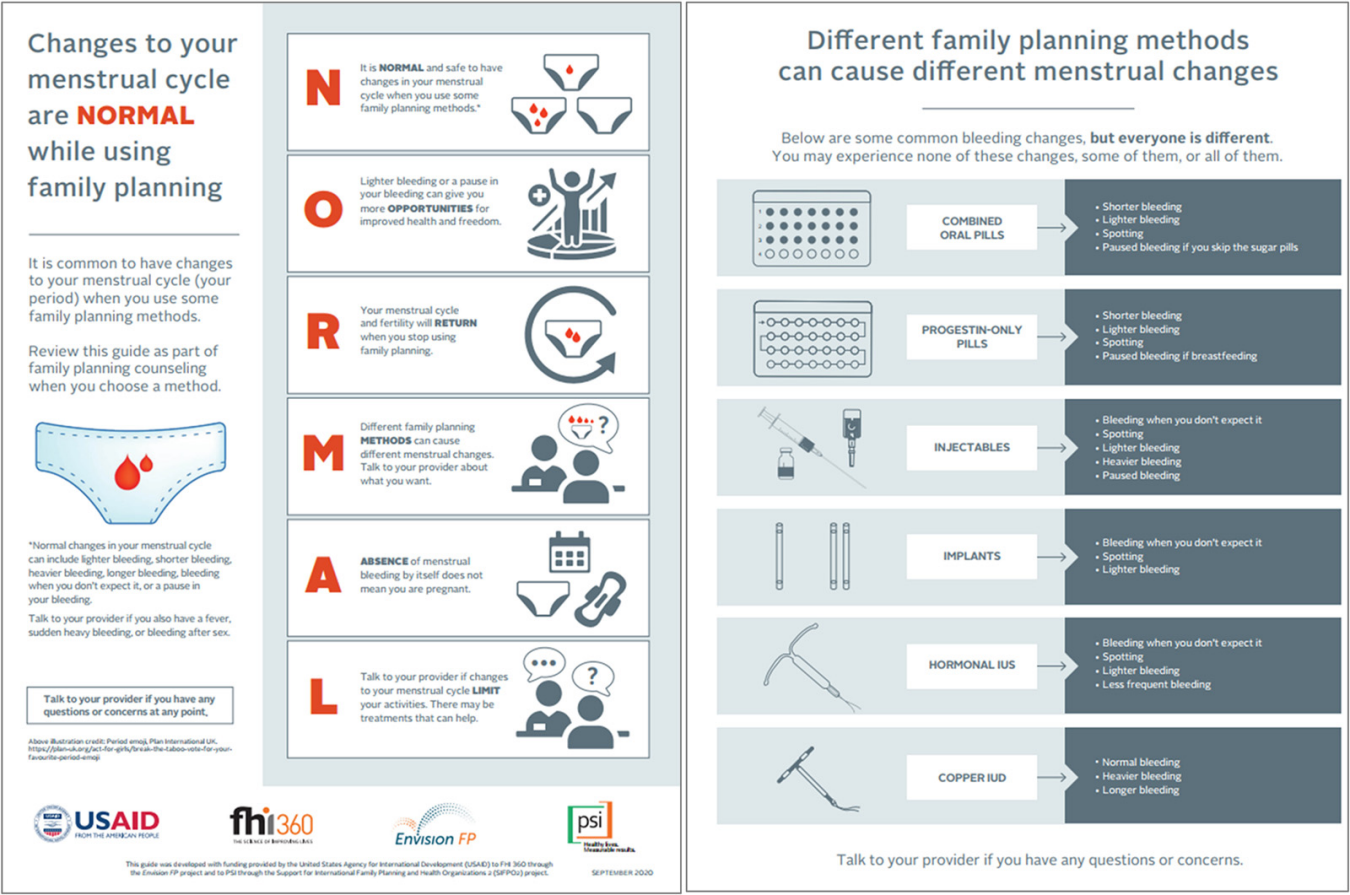
Original Community-Based NORMAL Job Aid

A recent review of FP counseling materials commonly used in international settings showed that these resources often do not adequately address common concerns and questions about CIMCs and often fail to highlight potential advantages of reduced or paused bleeding.^11^ In response, a facility-based version of the NORMAL job aid (also known as a counseling tool) was developed to address this gap. The job aid uses a simple mnemonic device “NORMAL”—Normal, Opportunities, Return, Methods, Absence of Menses, and Limit—to prompt health care providers to include 6 key counseling messages about CIMCs to address typical concerns and questions women often have (Figure 1).^11^ The NORMAL job aid was included in a counseling approach called Counseling for Choice (C4C) that was evaluated in private and public clinics in Malawi. Results showed that women counseled using C4C (including NORMAL) were more likely to report that their provider told them about what side effects to expect and how to manage them compared to those not receiving the intervention. Women counseled with C4C were also less likely to say that experiencing side effects would cause them to discontinue their chosen method (unpublished). Following this evaluation, several countries expressed interest in adapting and using the NORMAL tool. In addition, stakeholders from several countries, including Kenya, expressed the need for a lower literacy version of NORMAL that would be appropriate for use in community-based programs.

### Kenya Context

In Kenya, nearly 60% of currently married women use a contraceptive method, but 31% of FP users discontinue a method within 12 months of starting use.^12^ The modern methods most used by married women are injectables (26%), implants (10%), and oral contraceptive pills (8%).^12^ The most common reason for discontinuation is side effects and health concerns.^12^

In Kenya, the most common reason for contraceptive method discontinuation is side effects and health concerns.

Community health volunteers (CHVs), Kenya’s community health worker cadre, are not paid employees but are volunteers engaged by the Department of Health to provide services at the community level of the health care service delivery system. CHVs provide health messages at the household level, counsel families, trace those who are lost to follow-up, and provide a link to health facilities. In terms of FP services, CHVs provide information and counseling on all contraceptive methods using an FP counseling chart (job aid); can distribute progesterone-only pills, combined oral contraceptive pills, and male and female condoms; and refer clients to health care providers for other FP methods using a standard Ministry of Health referral form.

This case study was conducted within the Afya Uzazi project, which FHI 360 led in Nakuru and Baringo counties in Kenya with funding from the U.S. Agency for International Development (USAID). The project aimed to increase access to quality integrated health services, including strengthening the provision of FP services at facility and community levels. As part of this, technical support was provided to enhance CHV trainings, increase supportive supervision for CHVs, and improve data tracking and reporting. Afya Uzazi worked in 6 subcounties across the 2 counties.

We field-tested an adapted, 2-page, community-based version of the NORMAL job aid that is intended to guide CHVs on how to counsel women about CIMCs that are commonly experienced with the use of hormonal contraceptives and the copper IUD. This case study sought to understand how the job aid was used, the challenges faced in using it, and recommendations on how to improve the job aid. Data from this study were used to revise the job aid, develop recommendations for incorporating the job aid into FP counseling sessions, and facilitate clients’ use of the job aid during and after method selection and use.

## METHODS

### Study Setting

This study was conducted in the South Rift Valley region of Kenya in the Kuresoi North subcounty in Nakuru county and the Mogotio subcounty in Baringo county. These subcounties were purposively chosen in consultation with the county health management teams because of the large number of CHVs engaged in the Afya Uzazi project compared to other subcounties. Study staff collaborated with the departments of health in Baringo and Nakuru counties and Mogotio and Kuresoi North subcounties throughout the study to elicit their support and contributions to the study design, study implementation, and content of the community-based job aid. The facility-based version of the NORMAL job aid had not been introduced in the Afya Uzazi–supported health facilities in these counties.

### Study Design

We conducted a 2-phased qualitative descriptive study in the 2 subcounties. During the first phase, we gathered feedback on the job aid’s comprehensibility, acceptability, usability, and recommendations for improvement. We obtained this feedback through interviews with samples of CHVs, women who were current users and nonusers of hormonal contraceptive methods or the copper IUD. Figure 1 shows the original English version of the job aid that was used during the phase 1 interviews.

**FIGURE 2:**
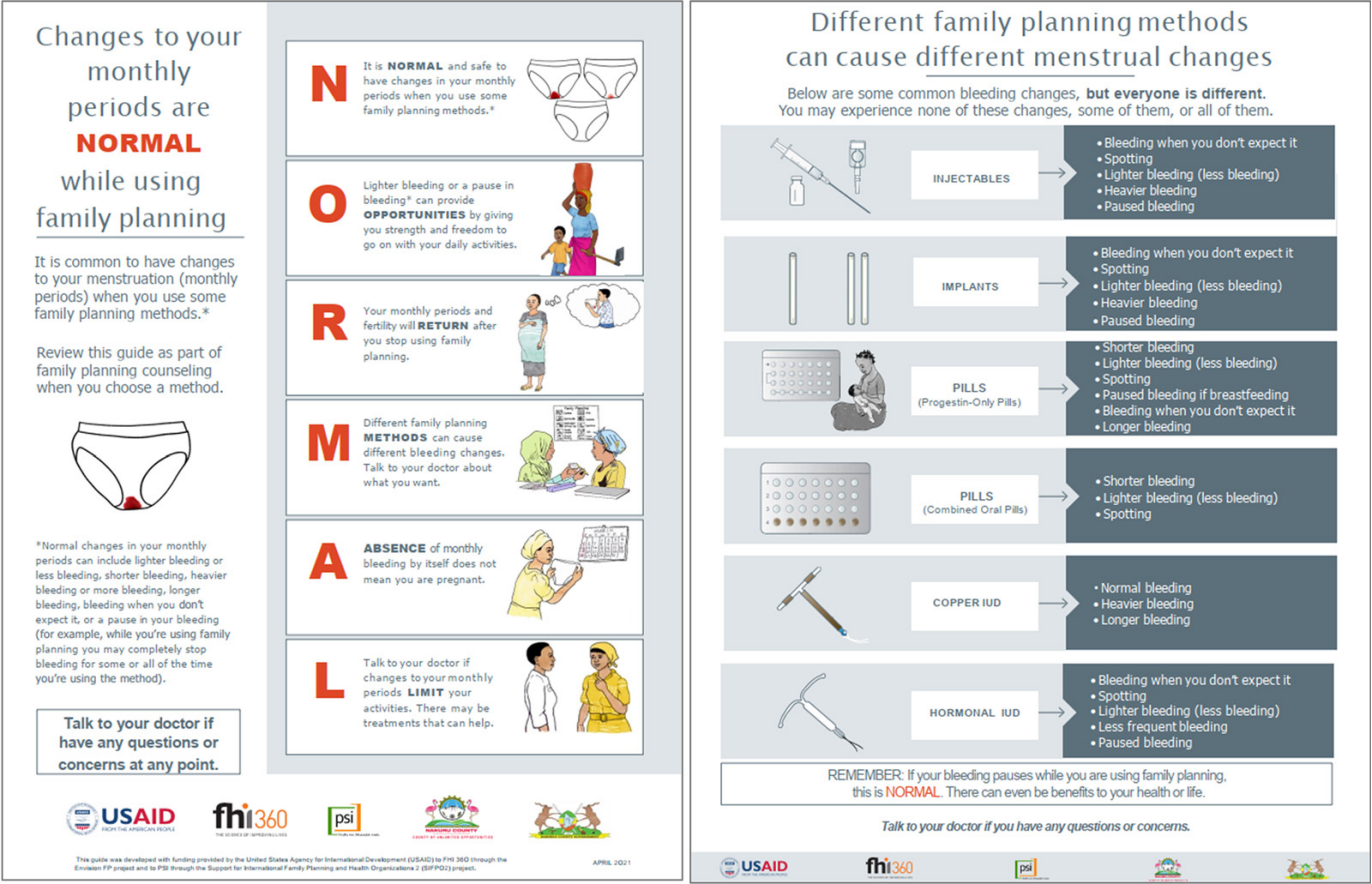
Community-Based NORMAL Job Aid Post-Phase 1

The job aid was translated into Kiswahili, and both the English and Kiswahili versions were discussed during the interviews. Based on the phase 1 feedback, we revised the job aid and used it in phase 2 of the study to field-test the job aid with select CHVs in the 2 subcounties. The field test in phase 2 aimed to understand how the job aid was used, the challenges faced in using it, and recommendations for improvement. Figure 2 shows the revised English job aid that was used in phase 2. CHVs were trained on the revised job aid and given English and Kiswahili copies of the job aid to use with clients. Six to 8 weeks after the training, we interviewed the CHVs about their experiences using the job aid and asked them to provide feedback on its utility, challenges in using it, and further recommendations to improve the job aid. We report on the results of the phase 2 field test.

### Sample Selection

As of September 2020, in the 6 subcounties supported by the Afya Uzazi project, there were 344 CHVs in Nakuru county and 596 CHVs in Baringo county. Every month, each CHV counsels an average of 12 FP clients; however, there is a wide range of client loads, with some CHVs counseling approximately 6 clients per week. In terms of literacy, CHVs are required to read and write basic English and Kiswahili.

Sixteen CHVs were recruited by Afya Uzazi program staff who already had an established working relationship with the CHVs to field-test the revised job aid. Afya Uzazi staff purposively selected CHVs from the 2 subcounties who had high FP client loads in the year before the study so the CHVs could potentially use the job aid with more clients before they were interviewed 6–8 weeks after the tool had been distributed. Purposive sampling, which is a nonprobability sampling design, involves the intentional selection of study participants so that information-rich cases (e.g., people who are knowledgeable or have experience about the phenomenon of interest) can provide information on the study objectives. Purposive sampling is best used when small sample sizes can help understand human perceptions, behaviors, and contexts for in-depth analysis.^13^ We included up to 8 in-depth interviews with CHVs for each county because research has shown that to be sufficient for thematic saturation.^14^

We estimated that the selected CHVs each counseled approximately 6 clients per week (totaling 36–48 clients counseled by each CHV using the job aid before the interview). Other eligibility criteria required the CHVs to be aged 18 years or older and assigned by the Ministry of Health to provide FP counseling to clients in Mogotio subcounty or Kuresoi North. CHV participants who participated in the study in phase 1 were also eligible to participate in phase 2. Two CHV participants (1 from each subcounty) participated in both phases. The CHVs gave their oral consent before the start of the interviews. There were no refusals to participate, and no participants dropped out of the study.

### Data Collection

CHVs were trained to use the revised job aid during an in-person training that lasted 4 hours. The training was led by an experienced female FP trainer (EW) in English and Kiswahili, depending on the preferences of the participants. The trainer who had been hired as a consultant for the study did not have any relationship with the CHVs before this study and was also an experienced qualitative interviewer with a master’s degree in anthropology and had worked on FP research. Eight CHVs were trained in Kuresoi North on April 26, 2021, and the other 8 CHVs were trained in Mogotio on April 28, 2021. The training addressed the topics outlined in Box 1. At the end of the training, the CHVs were given 100 copies of the job aid (80 in Kiswahili and 20 in English) to use with their clients. Additional copies were given to the reproductive health coordinator in both subcounties to distribute to the CHVs when needed.

BOX 1 **Topics Covered in Training**

- Family planning methods review
- Menstrual cycle review
- Overview of contraceptive-induced menstrual changes
- Introduction to the NORMAL job aid
- Practice with the job aid
  - Answering difficult questions
  - Dispelling myths
  - Addressing common issues such as menstrual stigma, infertility concerns, and misconceptions about how contraceptives work in the body
- Action planning
- Training evaluation

All 16 CHVs who were trained on the tool were invited to participate in an in-depth interview 6–8 weeks after the training. The trainer conducted the interviews in English or Kiswahili at a private location that was convenient and agreeable to the participants. The interviews averaged about an hour each and were audio-recorded. The interviewer took notes during the interview. For interviews conducted in English, the interviewer and a research assistant typed verbatim transcripts. For interviews conducted in Kiswahili, the interviewer and research assistant simultaneously translated and transcribed the audio-recordings into English. The interviews explored several topics, including if and how the CHVs used the job aid, any challenges they experienced, and further suggestions for improving the job aid. A short questionnaire was used to collect participants’ demographic information (e.g., age and education). Demographic data were entered into a Microsoft Excel spreadsheet.

### Data Analysis

We used a 2-step rapid qualitative analysis method to analyze data from the interviews.^15^ For the first step, a data analyst (CW) developed a structured template of a table using Microsoft Excel, which included columns for the questions from the interview guide and illustrative quotes. The analyst populated the template by reviewing and extracting data from each transcript, summarizing the data for each question, and including relevant illustrative quotes.

During the second step, the same analyst consolidated the summaries from the first step to identify commonly occurring themes (Supplement 1) and supporting quotes. The analysis focused on summarizing the CHVs’ experiences using the job aid with their clients and their recommendations to improve the job aid. Qualitative data from the 2 subcounties were analyzed together.

For the demographic questionnaire, the analyst conducted basic descriptive analyses (i.e., frequencies) to summarize the demographic data by subcounty. After data analysis was completed, data dissemination meetings were conducted in each of the 2 counties with all the CHV participants and key stakeholders, including county health department staff and partner organizations. During these meetings, study participants voluntarily provided feedback on the use of the job aid that was consistent with the findings.

### Ethical Approval

This research was reviewed and approved by the Scientific and Ethics Review Unit at Kenya Medical Research Institute in Kenya and FHI 360’s Protection of Human Subjects Committee.

## RESULTS

All 16 CHVs who were trained on the job aid were interviewed. The Table shows the sociodemographic characteristics of the participants for each subcounty. Seventy-five percent of the participants were female. The mean age of the Kuresoi North participants was slightly higher than the mean of Mogotio participants (47 years versus 43 years). While the participants in Kuresoi North were evenly split between those having some secondary and those completing secondary education, the participants in Mogotio had more diverse educational attainments. Mogotio participants had more years of FP counseling experience compared to participants from Kuresoi North, though the latter group counseled more clients the previous month (58 clients versus 32 clients).

**TABLE.** Sociodemographic Characteristics of CHV Participants, by Subcounty

	**Kuresoi North (n=8)**	Mogotio (n=8)
Median age (range), years	47.5 (34–56)	44.5 (30–52)
Sex, no. (%)		
Male	2 (25)	2 (25)
Female	6 (75)	6 (75)
Education, no. (%)		
Primary school/equivalent	0 (0)	1 (13)
Some secondary/equivalent	4 (50)	2 (25)
Secondary/equivalent	4 (50)	2 (25)
Postsecondary/University	0 (0)	3 (38)
FP counseling experience, years, no. (%)		
1–3	1 (12)	0 (0)
4–6	2 (25)	1 (13)
7–9	3 (38)	3 (38)
≥ 10	2 (25)	4 (50)
FP clients counseled in past month, median (range)	50 (20–100)	31.5 (19–50)

Abbreviations: CHV, community health volunteer; FP, family planning.

### Counseling on CIMCs Before the Introduction of the Job Aid

All but 1 CHV participant said that they did not have knowledge of CIMCs before using the job aid. As the CHVs quotes from both Mogotio and Kuresoi North demonstrate, without this knowledge, CHVs would refer clients who had problems to the health facility.

*Before I came for the training, I used to face the same questions as those I faced after I came for the training but before I came for the training, I used to refer to the health facility because I did not have enough knowledge to educate them.* —CHV, Mogotio

*In those cases, we used to refer them to the doctor, we did not have the knowledge of educating people that if you get this it is like this, we did not have that knowledge. But if someone comes to you or you go to them and they tell you that I used the injections and I bled the whole week, I would tell them to go to the hospital because that is a case where the doctor will be able to help you.* —CHV, Kuresoi North

A CHV with prior knowledge of CIMCs said that she had limited training on this topic that was not as detailed as provided in the NORMAL job aid training.

### Use of the Job Aid After Study Training

All CHV study participants reported using the job aid immediately after the training until they were interviewed, with all their FP clients, and each time they conducted FP counseling. The participants said they usually set aside 2–5 days a week for FP counseling, but they also conducted impromptu counseling outside of the set days. The CHVs said they used the job aid during their routine FP counseling and information-sharing sessions, which they conduct at households, workplaces, farms, churches, community meetings (e.g., chief baraza [public assembly], merry-go-round [savings group] meetings, and other women’s meetings), on the road, and during health talks at the health facility. Participants reported using the job aid with mostly women of reproductive age and older women, as well as men, to gain their support and to access women for FP counseling.

### What CHVs Liked About the Job Aid

All participants said that they liked the job aid and that it helped them provide better FP counseling to their clients. CHVs noted that the job aid and training gave them new knowledge, making their work easier.

*We have passed through various trainings, but we have never passed through a training like this one. We have been trained yes on FP, but we have never been trained on something like the job aid.* —CHV, Mogotio

When asked how the job aid made their work easier, participants noted that the job aid provided structure for counseling, explanations about the different FP methods, including those that are not commonly used (e.g., IUD and oral contraceptive pills for breastfeeding mothers) and their corresponding CIMCs, and a visual aid to assist with explanations of the different FP methods.

*It made my work easy because they not only listen to me counseling them using the job aid, they also see the illustrations of the messages or the methods on the job aid. When I am talking to them about pills, they can see what I am talking about. And I explain to them how it is used so that made my work easy.* —CHV, Kuresoi North

Participants noted that the job aid made the work easier by providing structure for counseling, explanations on FP methods and their CIMCs, and a visual aid.

In addition to increasing CHVs’ knowledge, the job aid increased their confidence in counseling about CIMCs.

*It was a confidence boost. Already I had gone for the training [referring to the job aid training], and I also had the job aid, and it was really helping me, and it was not like the time when I was just talking to them [clients]. You know at that time there are some things that you were guessing but with this job aid, and I have gone for the training, I have copies of the job aid, you know now these are things that are helping me.* —CHV, Mogotio

According to the CHV participants, the job aid enabled them to reach more people.

*Before it was like we were in darkness; light was there but it was still dark, but once they [clients and community members] started knowing, they came, all of them, the educated and uneducated and the interesting thing is that all of them want to learn*. —CHV, Kuresoi North

The participants also said the job aid assisted with addressing misperceptions about FP. CHVs mentioned several examples of myths and misperceptions that the job aid helped them address (Box 2).

BOX 2 **Examples of Client Misperceptions That Community Health Volunteers Reported the Job Aid Helped Them Address**

- A woman is cursed because of contraceptive-induced menstrual changes.
- When one uses family planning, they will always bleed a lot.
- If one experiences spotting, they will never give birth.
- If one uses the pill, then the contraceptive will attach itself to the womb.
- The ingredients in family planning will pile up in the body and therefore it is necessary to take herbal medicine to clear out those ingredients.
- The intrauterine device will disappear into the body once inserted.

### How Clients Used the Job Aid

According to the CHV participants, clients used the job aid to select an initial method or switch to another FP method.

*The clients say, “The job aid is good because if you want to stop using a method you look for another method in the job aid.”* —CHV, Mogotio

Other CHV participants described clients using the job aid to choose an FP method that suited them in terms of possible menstrual bleeding changes. For example, if clients did farm work, they may have preferred an injectable so that their periods were paused, and they would have the strength to do manual labor.

*Some women say that they would rather have injectables because they can have paused bleeding. Because some would argue that they will be fatigued during their monthly periods and not have strength to look for short-term farm work.* —CHV, Kuresoi North

Other clients became “ambassadors” by spreading the information to additional community members.

*Some were using it…even others were ambassadors who would tell many more and you would find when I go to the forest…we usually cultivate in the forest … you find them telling me “[Respondent's name withheld], I have heard about your teachings, and they are not bad so when I get a chance I will come to your home,” so even others come here on their own.* —CHV, Kuresoi North

### What CHVs Report Clients Liked About the Job Aid

According to the CHV participants, clients viewed the job aid as an “eye-opener” because it was the first time they learned about CIMCs, and the information was helpful in reducing husband-wife conflicts about bleeding changes.

*When they saw the bleeding thrice a month, they would think that it is a curse. But now if they see it, they know it is normal… and the husband in the house knows that it is not a curse because it was mostly conflict with the husband. Because you find that someone has had their menses 3 times in a month until she loses her respect in marriage. But these days the men understand that it is because of the method and so conflicts in the house have reduced.* —CHV, Kuresoi North

CHVs reported that clients viewed the job aid as the first time they learned about CIMCs.

CHV participants also noted that for those who had not started using FP yet, it was good to learn about the information on the job aid so that if they experienced those changes when they started FP, they already knew about it. Some clients asked the CHVs why they were not given the job aid and counseled on menstrual bleeding changes before they started using FP.

*I spent a lot of time there because she [client] has encountered challenges, and she wanted to know and ask me where this job aid was when she was being counseled before about the method. So I told her the job aid was not there at that time and it was recently introduced, then she asked which month the job aid was introduced, and I told her just recently. They understood and said if it was there from the beginning, she would not have gone through all the problems and the worries. —*CHV, Kuresoi North

### Offering the Job Aid to Clients

All CHV participants reported that they offered a copy of the job aid to most clients to take with them and that most clients took the job aid. Some clients asked to take additional job aids so that they could share them with friends. According to the CHVs, clients with low literacy took the job aid home so that they could ask their husbands or friends to read to them.

However, not all clients took the job aid with them. CHV participants reported that some clients did not take it because they were using FP discreetly and therefore had to hide their FP use from their husbands, parents, and others in their homes. After reading the job aid during the counseling session, some clients said they understood and did not need to take it with them.

CHVs said that they and their clients liked that the job aids were in both languages. According to the CHV participants, more clients preferred the Kiswahili version to the English version. However, having the English version was important because those who preferred it said it was easier for them to understand because they only knew the name of the FP method in English.

*Yes, English was easy to understand but this was not the case for everyone, some preferred Kiswahili because it was easy for them to understand, others preferred English and would find Kiswahili difficult to read and understand.* —CHV, Kuresoi North

### Impact of Job Aid on the Community

Most CHV participants reported that using the job aid increased their status in the community by making them more respected, trustworthy, and well-known. Participants noted that clients who have been counseled will now refer their friends and family to the CHV, and other CHVs who were not part of the study are referring their clients to see them. A clinical officer also requested several copies of the job aid from a CHV participant. Some CHVs noted that community members also are now going to them for FP advice when in the past, they did not.

*It has helped me because I have become known even more than before because even though I was known as a CHV but now … I am known as a mini-doctor of the village although I do not have medicine… because when I used to walk around, I would give them the job aid and they appreciate that someone is teaching and educating them… I am well known and famous.* —CHV, Kuresoi North

Four CHV participants said they trained other CHVs in their area on their own initiative so that they could use the job aid with their clients.

*Yes, and even other CHVs were asking me why I just went alone [to the job aid training] and without them. I also gave the CHVs these job aid materials, we are 10 CHVs and I gave them 1 job aid each, for them also to spread the information to every village, you know we are from different villages…They are now spreading the information. Since I came back from the training, FP practice in this area has increased.* —CHV, Kuresoi North

### Whether Job Aid Messages Have Changed Clients’ Attitudes Toward Using FP

According to the CHV participants, counseling with the job aid encouraged clients to start or return to using FP because their misperceptions about FP were addressed and they now understood that CIMCs were typically normal. Participants also described several men in their communities who changed their minds about FP and allowed their wives to start using FP after reading the job aid or after meeting the CHV.

*At first, he was thinking that his wife was sick, that every time he would touch his wife, there was bleeding. So, he said that he almost left his wife saying that this woman is sick, and she is not willing to share this with me. [Laughter] … So, he wanted to leave but bit by bit he told me that I had helped him tremendously and that he now realized that the bleeding is normal.* —CHV, Kuresoi North

CHV participants reported that counseling with the job aid encouraged clients to start or return to using FP because their misperceptions about FP were addressed.

According to the CHV participants, men in their communities would seldom see a CHV regarding FP, but now men were coming to the CHVs to learn more about FP because they had heard about or read the job aid from others.

*Yes, they [men] are continuing to change because when I give them this [job aid] to go and read, they give the information to their friends. So, their friends will say “this is not right since my wife is not thinking about using FP, I am the one who will go and inquire.” In this place I have met with 2 men, and they have told me that they have had enough children… He gave me permission to talk to the wife to choose what she will be using.* —CHV, Kuresoi North

### Whether the Job Aid Messages Helped Clients to Continue Using FP

According to the CHV participants, clients said that they were worried about CIMCs but now they knew these changes are normal.

*It has helped them [clients] because initially they were worried but when I educated them, they were satisfied, and they continued using [FP] without any fear.* —CHV, Mogotio

The CHV participants further specified, for women who had stopped bleeding, they recognized they could continue to work or go on trips, to church, or the market without worrying about managing their menstruation, and some noted they could either save or spend the money usually spent on buying menstrual pads in other ways.

*Those that do not receive their monthly periods have said that they are okay because the money that they used to spend on buying pads are now spent on merry-go-rounds [savings group] or on other things in the house. —*CHV, Kuresoi North

The participants also said that clients realized that the CIMCs they experienced depended on the FP method they were using and if they experienced heavier or longer bleeding, they knew that they could switch to other methods. According to some CHV participants, several clients switched methods for this reason.

*But the other day when I was educating them using the job aid, she told me that since now she knows when she finishes the 3 months injectable, she will go for the 3 years implant because now she knows about it. Because she was thinking that somebody will have periods on a daily basis when they use the implant but because [she now knows] you can have paused bleeding, she preferred the one for 3 years.* —CHV, Mogotio

### Suggestions for Improving the Job Aid

Most of the CHV participants did not have any suggestions for improving the job aid during Phase 2 of the study.

*“The only thing is to thank you for your time and for this training that you gave us because if it were not for that, I wouldn’t be seated here and the knowledge I got, I wouldn’t have. And the opportunity that this job aid has given me, I wouldn’t have received because it has saved time and made work easier.”* —CHV, Kuresoi North

However, some participants suggested adding definitions for each type of menstrual bleeding (“spotting,” “heavier bleeding,” and “paused bleeding”) because clients may not have known the terms or may have had a different definition of the terms. Another suggestion was to explain where the menstrual blood goes if a woman missed her period. A few CHVs provided formatting suggestions, such as to translate the job aid into additional local languages, print the job aid as a booklet so that it could not be easily lost or torn, create a poster with the information so that it could be hung up in a room at the health facility, and promote the job aid messages on social media. Though interview questions were purposely worded in an open-ended manner, we did not hear of any negative unintended consequences of the job aid during the interviews.

**FIGURE 3:**
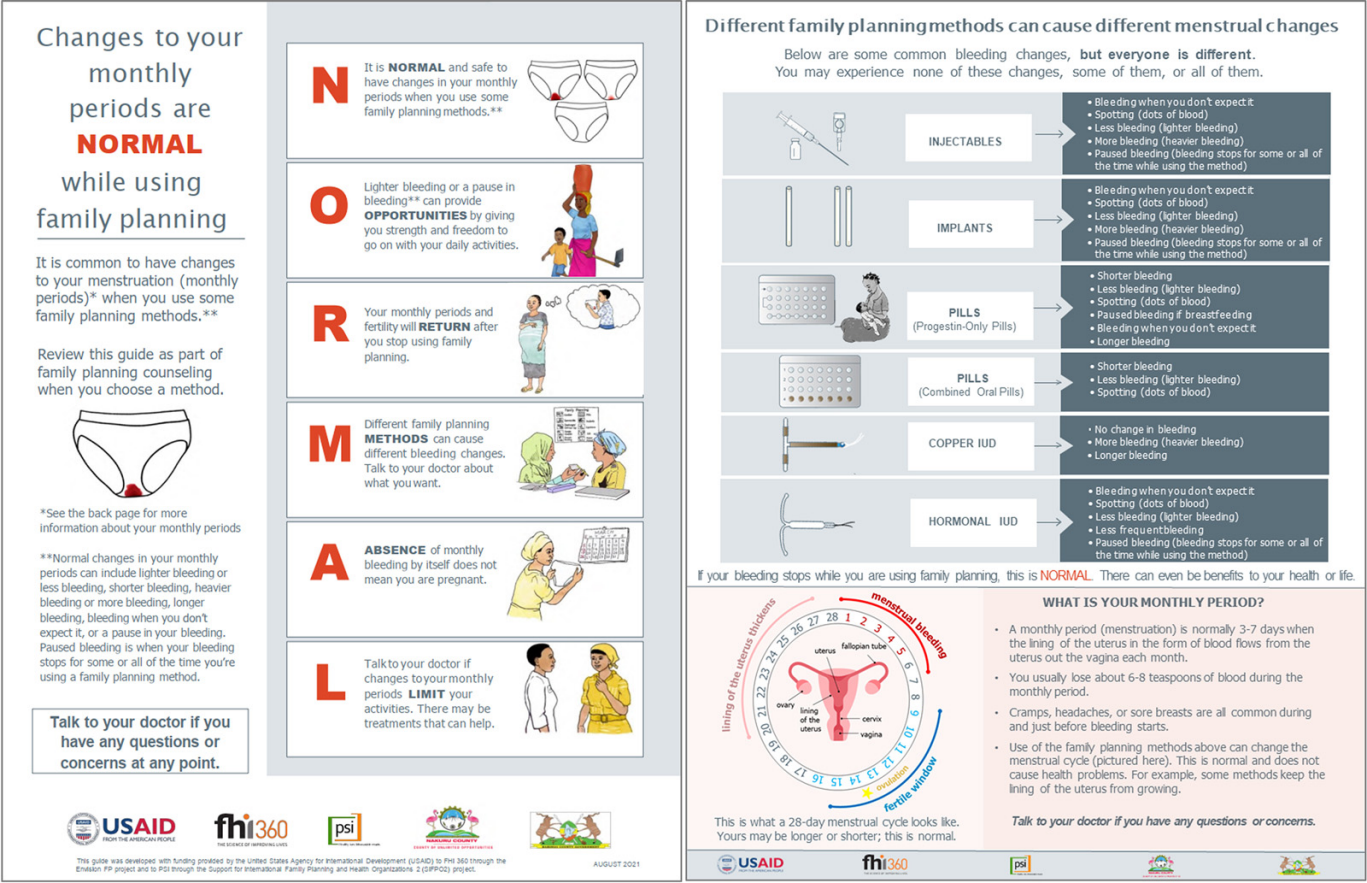
Community-Based NORMAL Job Aid Post-Phase 2

In response to this feedback, we made several changes to the job aid. First, we modified the language describing bleeding changes to define these terms and make them more descriptive and easier to understand. We also included additional basic information added about the menstrual cycle to enhance understanding and explain “where the menstrual blood goes” if a client missed her period. Figure 3 shows the final version of the job aid in English. Supplement 2 includes the Kiswahili version.

The CHV participants provided other suggestions for improvement that were not related to the job aid but indicated areas where CHVs could benefit from future training. For example, participants felt that each FP method should be covered in more detail during the training. They especially expressed a desire for more information about the IUD (e.g., size and insertion process). Participants felt that job aids should be developed for other FP methods, such as exclusive breastfeeding and the counting beads method, and to explain non-CIMC side effects for each FP method. The participants also said they would like to carry FP samples rather than just pictures to use during education and counseling for easier understanding and for clients to learn the actual size of the contraceptive.

## DISCUSSION

We found the revised NORMAL job aid was highly acceptable among a small sample of CHVs included in the study in 2 subcounties in Kenya. We learned that it was feasible for these CHVs to use the job aid during their FP counseling sessions, and the CHVs reported that the job aid increased the effectiveness of their counseling. This is similar to a variety of research in the field of FP showing the importance and effectiveness of job aids in enhancing FP counseling, especially for CHVs.^16^^,^^17^^,^^18^ The job aid appeared to increase the effectiveness of their counseling primarily by reducing clients’ concerns about CIMCs and helping clients select or switch FP methods. This is a significant finding because, while the current literature identifies a need for more and better counseling tools that address CIMCs,^2^^,^^3^ there is little research that has been conducted on the content needs, importance, and effectiveness of CIMC job aids and counseling. The original facility-based version of the NORMAL tool was informed by feedback and experience from private- and public-sector health care providers in Haiti and Zambia but was not formally tested for effectiveness.^11^ One study that does support the claims that CIMC counseling is effective was identified through a literature review examining strategies for improving adherence and acceptability of hormonal contraceptive methods. Reviewers found that clients who received repeated, structured information about injectable contraceptives were less likely to discontinue due to menstrual disturbances.^19^

The job aid appeared to increase the effectiveness of their counseling primarily by reducing clients’ concerns about CIMCs and helping clients select or switch FP methods.

Enhanced CHV counseling made possible by use of the NORMAL job aid is important because it may reduce clients’ trips to the facilities if CHVs can answer women’s questions about CIMCs in community settings. Reduced visits to the facilities may save women time and money as well as reduce the burden on health facilities. This claim is supported by 2 FP High Impact Practices (HIPs). First, there is a HIP brief on community health workers (CHWs) that provides substantial evidence in support of the statement that “CHWs are particularly important to reducing inequities in access to services by bringing information, services, and supplies to women and men in the communities where they live and work rather than requiring them to visit health facilities, which may be distant or otherwise inaccessible.”^20^ In addition the HIP strategic planning guide on task-sharing cites several research studies showing improved and more equitable access to contraceptive services and an increase in health system efficiencies when clients can access care at several delivery points, including from CHWs/CHVs.^21^

Further, CHVs’ use of the job aid appears to diffuse information about CIMCs beyond their FP clients into the community, including among male partners. The information in the job aid appeared to provide reassurance about CIMCs to male partners and improve relationship dynamics. There is research showing the power of informal networks in spreading health information to a variety of stakeholders, including to male partners.^22^^,^^23^ The literature also provides evidence that CHVs play an important role in the dissemination of health information in communities.^24^^,^^25^ However, a rapid review of the literature does not identify any study showing the use of a job aid and the role of CHVs in diffusion of FP information to male partners specifically. This finding is important because the job aid may increase male partners support of contraceptive use and be a tool for engaging men in FP—an important strategy for improving reproductive health and gender equality.^26^^–^^29^

The NORMAL job aid also appears to increase women’s self-awareness that CIMCs are common and not harmful to one’s health. Further women can use the job aid to reassure themselves of these messages which may improve contraceptive continuation rates.^30^ The World Health Organization defines self-care as “the ability of individuals, families, and communities to promote health, prevent disease, maintain health, and cope with illness and disability with or without the support of the health provider.”^31^ Self-care spans a range of practices including self-awareness, self-testing, and self-management. Self-care interventions are tools that support self-care. Given the growing interest in interventions and strategies that promote self-care within health systems, FP programs could benefit from a tool like the NORMAL job aid that promotes self-reassurance.

However, further research is needed to confirm the findings from this study, especially in understanding the best messaging and counseling interventions that will reassure clients about CIMCs to improve uptake and reduce discontinuation of hormonal FP methods and the copper IUD across a variety of contexts.

### Limitations

This study’s greatest limitation was its small sample size. Therefore, the study results may not be generalizable outside of the study setting. Another limitation is that the same individual who trained the CHVs to use the job aid also conducted the interviews with the CHVs; therefore, there is the potential for social desirability bias because the CHVs might have reported higher use and/or acceptability of the job aid to please the trainer/interviewer. We attempted to protect against this bias by stating at the beginning of the interviews that our goal was to improve the job aid and by having open-ended interview questions so that it was clear that we were seeking both positive and negative feedback. Despite these limitations, we found remarkably consistent themes across the interviews, and thus, reached thematic saturation with our small sample. Further, the high acceptability of the job aid described by the CHV participants was strongly supported by detailed explanations and examples which increases our confidence in the findings. Finally, this study only examined the job aid from the perspectives of the CHVs and did not include data collection with FP clients. Future research should include clients’ perspectives, include larger samples of CHVs, and measure the effectiveness of the job aid on uptake and continued use of hormonal FP methods and the copper IUD.

## CONCLUSION

To our knowledge, this is the first job aid of its kind that is intended to guide CHWs on how to counsel women about CIMCs. Based on the positive results of this field test, we recommend this job aid be tested and introduced on a wider scale in Kenya where the CHVs and clients share a similar dialect with those who participated in this study. Where audiences may differ, we recommend additional pretesting and adaptation of the job aid to ensure comprehension and acceptability. Providing clients copies of the job aid, as the CHVs did in this study, will likely increase diffusion of the information, but if this is not feasible for programs, then providing CHVs with a durable copy in Kiswahili (or any additional local language) and English to use during counseling sessions will still likely be beneficial.^32^ Though outside of the scope of the job aid, CHVs in the study setting would benefit from a refresher on FP methods, especially those methods that are not commonly used (e.g., IUD). Preliminary findings suggest that use of the job aid could help increase uptake and continued use of hormonal FP methods and the copper IUD. Further research is needed to evaluate the potential impact of broader use of the job aid.

## Supplementary Material

GHSP-D-22-00364-Supplement2.pdf

22-00364-Burke-Supplement1.docx

GHSP-D-22-00364-Supplement1.docx

22-00364-Burke-Supplement2.pdf

## Notes

**Peer Reviewed**

**First published online:** February 10, 2023.
