# Supplementary material for: A Field Test of the NORMAL Job Aid With Community Health Workers in Kenya to Address Contraceptive-Induced Menstrual Changes
Source: Glob Health Sci Pract. 2023 Feb 28;11(1):e2200364. doi: 10.9745/GHSP-D-22-00364 (PMC9972389; doi:10.9745/GHSP-D-22-00364)
Supplement: GHSP-D-22-00364-Supplement2.pdf [file GHSP-D-22-00364-Supplement2.pdf]

Mabadiliko ya Damu ya Mwezi zako ni **KAWAIDA** wakati unapotumia Njia za Kupanga Uzazi

Ni kawaida kuwa na mabadiliko ya damu ya mwezi* wakati unapotumia njia za Kupanga Uzazi.**

Angalia upya fomu hii ya kufanya kazi kama Moja ya vifaa vya ushauri wakati unapochagua njia ya Kupanga Uzazi.

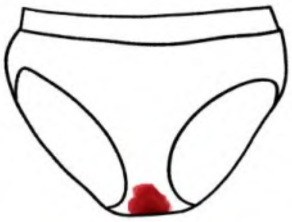

*Enda kwa ukuraza wa mwisho kwa maelezo zaidi kuhusu damu yako ya mwezi

** Mabadiliko ya kawaida katika damu ya mwezi ni kama vile Hedhi nyepesi (damu inayotoka kidogo tu), Hedhi za mda mfupi, Hedhi nzito (damu inayatoka sana), Hedhi za mda mrefu, Hedhi wakati hizitarajii, ama Hedhi kuacha kutoka kwa muda. "Hedhi kuacha kutoka kwa muda". Kusitisha kwa damu ya mwezi ni wakati damu yako ya mwezi inaposimama kwa muda au kwa wakati wote unapotumia mbinu za kupanga uzazi

Zungumza na Daktari wako kama uko na maswali yoyote ama wasiwasi wakati wowote.

N

Ni **KAWAIDA** na salama kuwa na mabadiliko katika Hedhi (damu ya mwezi) wakati unapotumia njia za Kupanga Uzazi.**

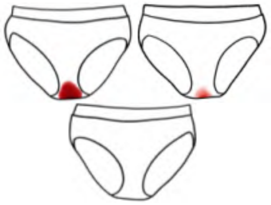

O

Hedhi (damu ya mwezi) inayotoka kidogo, ama Hedhi kuacha kutoka kwa muda** inaweza kutoa **NAFASI** kwa kukupa nguvu na uhuru kuendelea na shughuli zako.

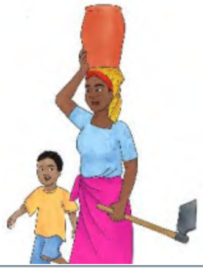

R

Damu ya mwezi yako na uzazi **ITARUDI** unapoacha matumizi ya njia za Kupanga Uzazi.

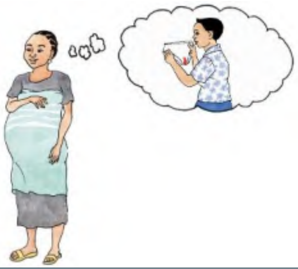

M

NJIA tofautitofauti za Kupanga Uzazi zinaweza kusababisha mabadiliko katika damu ya mwezi. Zungumza na Daktari wako kuhusu mahitaji yako.

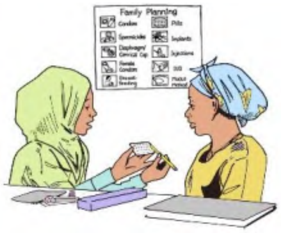

A

KUKOSEKANA kwa damu ya mwezi haimanishi kwamba umepata mimba.

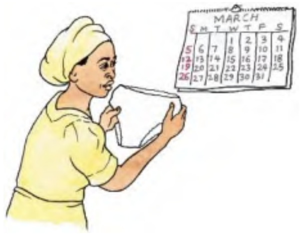

L

Zungumza na Daktari wako kama mabadiliko katika damu ya mwezi **HUZUIA** shughuli zako. Labda kunaweza kuwa na tiba zinazoweza kusaidia.

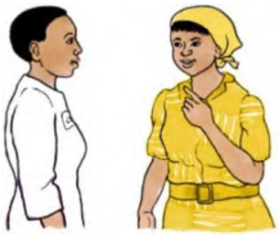

USAID FROM THE AMERICAN PEOPLE | fhi360 THE SCIENCE OF IMPROVING LIVES | psi | NAKURU COUNTY COUNTY OF UNLIMITED OPPORTUNITIES | BARINGO COUNTY GOVERNMENT

Muongozo huu ulitengezwa kwa Ufadhili wa Fedha kutoka United States Agency for International Development (USAID) kwa FHI 360 kupitia mradi wa Mpango wa Uzazi wa Envision na kwa PSI kupitia kwa ushirikiano na mradi wa International Family Planning and Health Organizations 2 (SIFPO2)

AGOSTI 2021

Njia tofautitofauti za Kupanga Uzazi zinaweza kusababisha mabadiliko tofautitofauti ya Hedhi

Hapa chini kuna baadhi ya mabadiliko ya Hedhi ya kawaida, lakini kila mmoja ni tofauti. Labda unaweza kuhisi mabadiliko haya, baadhi yao, ama yote.

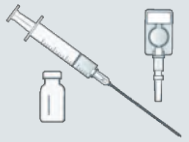

SINDANO ya Kupanga Uzazi →

- Kupata Hedhi wakati usipotarajia
- Madoa ama tone ya Hedhi
- Damu inayatoka kidogo (hedhi nyepesi)
- Damu inyatoka nyingi (hedhi nzito)
- Hedhi kuacha kutoka kwa muda (damu itaacha kutoka kwa muda au kabisa wakati unatumia njia)

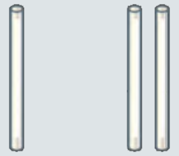

VIPANDIKIZI vya Kupanga Uzazi →

- Kupata Hedhi wakati usipotarajia
- Madoa ama tone ya Hedhi
- Damu inayatoka kidogo (hedhi nyepesi)
- Damu inyatoka nyingi (hedhi nzito)
- Hedhi kuacha kutoka kwa muda (damu itaacha kutoka kwa muda au kabisa wakati unatumia njia)

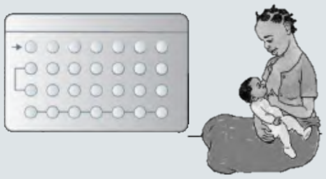

VIDONGE (Vya homoni ya Progestin pekee) →

- Hedhi ya mda mfupi
- Damu inayatoka kidogo (hedhi nyepesi)
- Madoa ama tone ya Hedhi
- Hedhi kuacha unaponyonyesha
- Kupata Hedhi wakati usipotarajia
- Hedhi ya mda mrefu

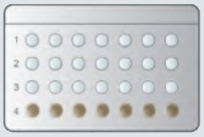

VIDONGE (Vyenye mchanganyiko wa Homoni) →

- Hedhi ya mda mfupi
- Damu inayatoka kidogo (hedhi nyepesi)
- Madoa ama tone ya Hedhi

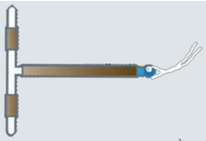

KITANZI cha IUD →

- Hakuna mabadiliko katika damu ya mwezi (hedhi)
- Damu inyatoka nyingi (hedhi nzito)
- Hedhi ya mda mrefu

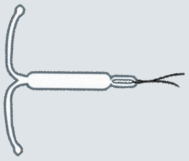

HOMONI za IUD →

- Kupata Hedhi wakati usipotarajia
- Madoa ama tone ya Hedhi
- Damu inayatoka kidogo (hedhi nyepesi)
- Hedhi isiyotoka mara kwa mara
- Hedhi kuacha kutoka kwa muda (damu itaacha kutoka kwa muda au kabisa wakati unatumia njia)

Kuwa damu yako ya mwezi ikiacha kutoka kwa muda wakati unatumia njia za Kupanga Uzazi, ni KAWAIDA.

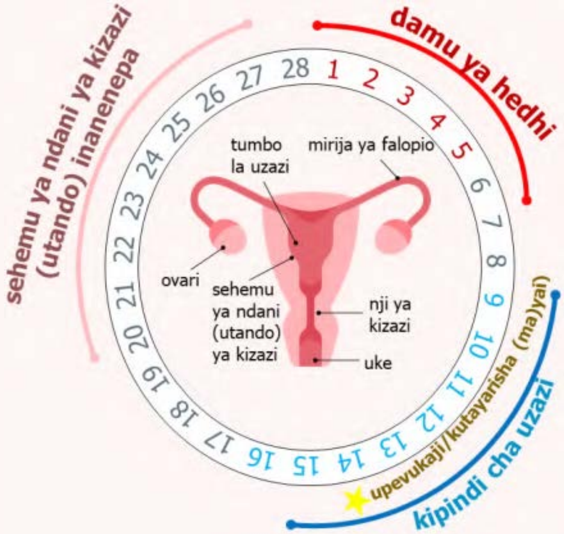

Hivi ndivyo mzunguko wa hedhi wa siku 28 unavyokaa. Yako inaweza kuwa refu au fupi: hii ni kawaida.

DAMU YAKO YA MWEZI NI NINI?

- Damu ya mwezi (Hedhi) hua ni siku 3 hadi 7 wakati sehemu ya ndani (utando) ya kizazi inayo kaa kama damu itokayo kwenye nyumba ya kizazi kupitia sehemu ya mwanamke ya siri kila mwezi.
- Huwa unapoteza takriban vijiko vya chai 6 hadi 8 ya wakati wa damu ya mwezi.
- Kuumwa na tumbo, kuumwa na kichwa, au uzito na maumivu ya matiti, ni kawaida wakati au kabla ya kupata damu ya mwezi.
- Matumizi ya mbinu ya kupanga uzazi iliyo hapo juu inaweza kusababisha mabadiliko katika damu ya mwezi (yenye imewekwa hapa). Kwa mfano mbinu zingine zinafanya sehemu ya ndani (utando) ya kizazi kukua na mbinu zingine.

Zungumza na Daktari wako kama uko na maswali yoyote ama wasiwasi wakati wowote.

USAID FROM THE AMERICAN PEOPLE
fhi360 THE SCIENCE OF IMPROVING LIVES
psi
NAKURU COUNTY
