# Supplementary material for: A Field Test of the NORMAL Job Aid With Community Health Workers in Kenya to Address Contraceptive-Induced Menstrual Changes
Source: Glob Health Sci Pract. 2023 Feb 28;11(1):e2200364. doi: 10.9745/GHSP-D-22-00364 (PMC9972389; doi:10.9745/GHSP-D-22-00364)
Supplement: 22-00364-Burke-Supplement1.docx [file 22-00364-Burke-Supplement1.docx]

**Supplement. List of themes and subthemes that emerged from community health volunteer in-depth interviews about using NORMAL job aid**

1. Use of job aid after training

- Whether job aid was used
- When CHVs started using the job aid

1. Experience using the job aid

- What CHVs liked about the job aid
  - What kinds of misconceptions exist about family planning that the job aid helped address
- What CHVs did not like about the job aid

1. Comprehension of the job aid

- What was easy to understand
  - Language used
  - Pictures used
- What was difficult to understand

1. How the CHV has been using the job aid

- Job aid has been used with clients when discussing what kinds of issues
- Types of clients CHV used the job aid with
  - Women
  - Men
- Places CHVs used the job aid at
- Other CHVs were also trained by study participants

1. Offering job aids to clients to take back with them

- Whether job aid was offered to clients to take back home with them
- Reasons for taking job aid home
- Reasons for not taking job aid home
- Whether low/non-literate clients took the job aid home with them and reasons why

1. Difference in counseling literate vs. low/non-literate clients
2. Counseling on menstrual bleeding changes prior to the job aid introduction

- Did not have the knowledge on menstrual bleeding changes in the past
- Had knowledge on menstrual bleeding changes in the past

1. How clients used the information from the job aid

- To choose or switch to another FP method before going to see the doctor
- Helped some to go see the doctor
- Others

1. Men have become receptive to discussing about FP
2. Feedback from clients about the job aid according to the CHVs

- What clients liked about the job aid according to the CHVs
- What clients thought about the English and Kiswahili version of the job aid
  - Clients preferred the English version and reasons why
  - Clients preferred the Kiswahili version and reasons why

1. Impact of job aid on the way CHVs provide counseling to clients

- Job aid has really helped them in their work
- Status in the community has been elevated

1. Whether job aid messages have changed clients’ attitudes towards using hormonal contraceptives or IUD
2. Whether the job aid messages helped clients to continue using FP methods even if they experienced bleeding changes
3. Challenges CHVs face in general when conducting FP counseling

- Barriers due to religion
- Barriers due to gender
- Barriers due to stock out
- Barriers due to community attitudes

1. Challenges CHVs face in using the job aid

- Language
- Counseling non-literate clients
- Others

1. Suggestions for improvement on the job aid

- Improvements needed for:
  - Pictures
  - Explanations
  - Language used
  - Size of job aid

1. Suggestions for improvement on the job aid training
2. Non-job aid related suggestions for improvement
3. Non-job aid related challenges that CHVs face
